# Androgen receptor decreases the renal cell carcinoma bone metastases via suppressing the osteolytic formation through altering a novel circEXOC7 regulatory axis

**DOI:** 10.1002/ctm2.353

**Published:** 2021-03-24

**Authors:** Dongkui Gong, Yin Sun, Changcheng Guo, Tzong‐jen Sheu, Wei Zhai, Junhua Zheng, Chawnshang Chang

**Affiliations:** ^1^ Department of Urology The First Affiliated Hospital of Soochow University Suzhou Jiangsu China; ^2^ George Whipple Lab for Cancer Research Departments of Pathology Urology, Radiation Oncology and The Wilmot Cancer Institute University of Rochester Medical Center Rochester New York USA; ^3^ Department of Urology Shanghai Tenth People's Hospital Tongji University School of Medicine Shanghai China; ^4^ Department of Orthopedics and Center for Musculoskeletal Research University of Rochester Medical Center Rochester New York USA; ^5^ Department of Urology Renji Hospital School of Medicine in Shanghai Jiao Tong University Shanghai China; ^6^ Department of Urology Shanghai General Hospital Shanghai Jiaotong University School of Medicine Shanghai China; ^7^ Sex Hormone Research Center China Medical University/Hospital Taichung Taiwan

**Keywords:** androgen receptor, bone metastases, ccRCC, circEXOC7, CSF1, DHX9, miR‐149‐3p

## Abstract

**Background:**

Clear cell renal cell carcinoma (ccRCC) has gender differences, with the androgen receptor (AR) linked positively with metastasis to the lung. Its linkage to ccRCC bone metastases (RBMs), however, remains unclear.

**Methods:**

In the current study, five human RCC and five RCC bone metastasis tissues were deeply sequenced using Arraystar human circRNA V2.0 microarray. We conducted gain‐of‐function screening in vitro and in vivo to elucidate the AR's role in the RBM. Loss/gain‐of‐function was also implemented to verify the roles of related non‐coding RNAs and proteins.

**Results:**

We uncovered that RBM also has a gender difference showing higher AR expression may be linked to fewer RBMs, which might involve suppressing osteolytic formation. Mechanism dissection indicates that AR can decrease the circular RNA EXOC7 (circEXOC7), expression via enhancing transcription of DHX9, a regulatory protein in circRNA biogenesis. The circEXOC7 can sponge/suppress miR‐149‐3p resulting in suppressing the CSF1 expression by directly binding to the 3′UTR region of CSF1 mRNA. Results from clinical epidemiological surveys also found that AR has a positive correlation with miR‐149‐3p and a negative correlation with CSF1 in AR‐positive ccRCC tissues. Preclinical studies with Balb/c nude mouse model also validated that targeting this newly verified AR/DHX9/circEXOC7/miR‐149‐3p/CSF1 signaling via altering circEXOC7 or AR could lead to suppressing the RBM progression.

**Conclusions:**

These data showed that AR/DHX9/circEXOC7/miR‐149‐3p/CSF1 signaling acts as a valuable feature in the bone metastasis of renal cancer, which may benefit in suppressing the RBM progression.

## Graphical abstract

AR is highly expressed in ccRCC tissues and low in RBM tissues.

AR/circEXOC7 is correlated with osteolytic formation of bone marrow‐derived macrphages.

AR can transcriptionally induce DHX9 to regulate circEXOC7 expression in ccRCC cells.

Both in vivo and in vitro data show that the novel ceRNA regulatory axis is involved in regulating RBM progression.

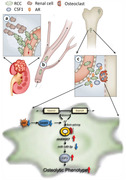

**Abbreviations**
AR: androgen receptor
AREs: androgen response elements
ccRCC: clear cell renal cell carcinoma
ChIP: chromatin immunoprecipitation assay
circRNA: circular RNA
miRNA: microRNA
RBM: RCC bone metastases
RIP: RNA immunoprecipitation

## 1 BACKGROUND

Renal cell carcinoma (RCC) is a highly malignant urological tumor, accounting for 4.1% of all new malignant cases, with 73,750 new cases and 14,830 deaths estimated for 2020 in the USA alone.^1^ A subset of patients had metastatic lesions when they were initially diagnosed,^2^ yet the specific mechanisms of RCC metastases are still unclear. Clear cell RCC (ccRCC) is the most common histologic subtype, is derived from the proximal renal tubule epithelial cells, and may be resistant to radiotherapies and multiple chemotherapies.

For RCC patients, 5‐year survival rates are near 93%, which may decrease to 12% for metastatic renal cell carcinoma (mRCC).^3^ The three most common sites of mRCC are lung, bone, and lymph nodes. The occurrence of RCC bone metastases (RBM) has been reported in 35–40% of patients with advanced RCC.^4^, ^5^ Approximately one third of RCC patients having received therapies for localized RCC may still develop metastases in distant sites.^6^, ^7^ Those RBMs mainly occur in the proximal extremities, pelvis, and spine, and nearly 80% of them are osteolytic lesions.^8^ Due to the linkage to the promotion of osteolysis, 74–85% of RBM patients may develop some skeletal‐related events, including pathological fractures, osteodynia, spinal cord compression, hypercalcemia, which impact severely the quality‐of‐life and even overall survival time.^5^ The complications that are initiated from those invasive RCC cells may disrupt bone remodeling and osteoblastic/osteolytic balance that result in pathological bone loss. The detailed mechanisms of how metastatic RCC cells alter the bone loss, however, remain unclear.^9^

The ccRCC has an obvious gender bias; this characteristic may be related to the differential expression of a certain sex hormone or its receptor.^10^ Early studies confirm that the androgen receptor (AR) is involved in the regulation of RCC initiation and progression.^11^, ^12^, ^13^ Moreover, AR could differentially impact the RCC metastases, with higher AR leading to increased lung metastasis yet decreased lymphatic metastases.^14^, ^15^ These contrasting differences indicate that AR may act in differentiated organ‐specific metastases’ manner. AR's role in the bone metastases, however, remains unclear.

As a kind of non‐coding RNA, the important role of circular RNA (circRNAs, ciRs) has not been confirmed until recent years.^16^, ^17^ Numbers of studies indicated that circRNAs might sponge microRNAs (miRNAs, miRs), code polypeptides, or directly bind to multiple proteins to alter the expression of some selective tumor‐related genes to impact the tumor progression.^18^, ^19^, ^20^, ^21^, ^22^ Also, circRNAs have been found to encode peptides as well as regulate transcription.^23^, ^24^, ^25^, ^26^ Their impacts on the RCC metastases, however, have not been fully examined.^27^

Here, we found AR may function via the DHX9/circEXOC7/miR‐149‐3p/CSF1 signaling to attenuate RBM, and targeting this signaling pathway with altered expression of circEXOC7 could suppress the osteolytic RBM.

## 2 METHODS

### 2.1 Clinical samples

We collected five ccRCC and five RBM samples from patients who underwent radical or partial nephrectomy and bone metastasis lesion resection in the Department of Urology, Renji Hospital. The clinical survey of patients and the tissue‐microarray including 59 specimens were based on the Department of Urology database. The fresh tissues were frozen in liquid nitrogen to protect the RNA from degradation within 30 min after resection. Human samples studies were approved by the ethics committee of the Ethical Committee and Institutional Review Board of the Renji Hospital Shanghai Jiao Tong University.

### 2.2 Cell culture

Human SW839, 786‐O, Caki‐1, ACHN, and HEK293T cell lines were obtained from the American Type Culture Collection (ATCC, Rockville, MD, USA). Human OS‐RC‐2 cell line was obtained from the National Infrastructure of Cell Line Resource (Beijing, China). Cells were cultured in a 5% CO_2_ humidified incubator at 37°C. DMEM and α‐MEM media supplemented with 10% fetal bovine serum and 1% penicillin and streptomycin were used to culture cells.

### 2.3 Plasmid‐inserted lentivirus preparation

Following the calcium chloride transfection method, HEK 293T cells were transfected with target plasmid (pLKO‐miR‐149‐3p, pLKO‐shCSF1, pLKO‐shcircEXOC7, pLKO‐shAR, pWPI‐AR, or pWPI‐circEXOC7) and envelope plasmids (psPAX2 and pMD2.G). After 2 days, the lentivirus media were collected, concentrated, and stored at −80°C. The sequencing of circEXOC7 was amplified by PCR and inserted into the empty pBSK (Addgene, MA, USA) vector as the pBSK_circEXOC7, then fragments digested and cloned to the lentiviral pre‐digest pWPI vector for expressing circEXOC7. To clone the circRNA sequencing from the pBSK_circEXOC7, the forward primer was 5′‐GTGAGGAATTTCGACATTT AAATTTAAAAGTGCTGAATTACAGGCG‐3′, and the backward primer was 5′‐TCCTGCAGCCCGTAGTTTTGCTGGGATTACAGGTGTGA‐3′. All oligo fragments' detailed information is shown in Table S1.

### 2.4 Proliferation and differentiation of bone marrow‐derived macrophages

RCC cells were transfected with virus. After 24 h transfection, the media from RCC cells were collected as conditioned media (CM). Bone marrow‐derived macrophages (BMMs) are primary cells, which were isolated from C57/BL6 male mice thigh bone marrow as described previously. Briefly, bone marrow was seeded into 24‐well plate for 72 h, incubated with parathyroid hormone (PTH) (100 ng/ml) in the presence of 30% CM, which was renewed every 48 h. BMM differentiation could be identified after 5–7 days incubation and tartrate‐resistant acid phosphatase (TRAP+) staining and counted with Image‐pro Plus (Media Cybernetics, MD, USA). The detailed protocol is included in *Supplementary Materials*. TRAP staining was used to identify osteoclast formation. Briefly, cells were washed with PBS for one time after removal of the culture media with a pipette. Then cells were fixed with 10% formalin at room temperature for 30 min followed by, PBS wash for three times, refixed with 95% ethanol: aceton (1:1) at room temperature for 1 min and air dried for 10 min. Cells were Trap stained in solution for 30 min at 37°C followed by PBS wash. 3‐[4,5‐dimethylthiazole‐2‐yl]‐2,5‐diphenyltetrazolium bromide (MTT) assay was used to analyze the proliferation of BMMs cells during co‐culture (using α‐MEM). MTT reagent was added and incubated for 2 h at 37°C, then Dimethyl Sulfoxide (DMSO) was added to dissolve the crystals before the endpoint of incubation, then measured the absorbance at 450 nm. Each sample was conducted in triplicate.

### 2.5 Quantitative real‐time RT‐PCR

Total RNAs were extracted with Trizol (Invitrogen, NY, USA), and then reverse transcribed into cDNA. Then quantitative real‐time RT‐PCR (qRT‐PCR) was performed to evaluate the mRNA expression of the target gene, which was normalized by GAPDH or β‐actin expression. All primers’ detailed information is shown in Table S1.

### 2.6 Western blot analysis

Proteins, measured with Rapid Gold BCA (Thermo Fisher Scientific, MA, USA) method, were separated on 8–12% SDS/PAGE gel and transferred onto PVDF membranes (Millipore Sigma, Germany). Non‐fat milk was used to block the membranes. Then the membranes were incubated with specific primary antibodies and horseradish peroxidase (HRP)‐conjugated secondary antibodies successively and then visualized with ECL system (Thermo Fisher Scientific).

### 2.7 RNA immunoprecipitation

Native RNA immunoprecipitation (RIP) was conducted following the published study. Briefly, lysis buffer supplemented with RNase inhibitor was used to lyse the cells, protein A/G beads added to the supernatant (10 mg) for 1 h, incubated with Argonaute‐2 (Ago2) antibody over 12 h at 4°C, then pre‐blocked beads added with BSA to the mixture and incubated for 2 h. The RIPA buffer pre‐mixed with protease inhibitor and cocktail RNase inhibitor was used to wash the complex three times. Trizol was used to extract total RNAs from the complex.

### 2.8 RNA fluorescent in situ hybridization

RNA fluorescent in situ hybridization (RNA‐FISH) analysis was used to visualize the RNA subcellular location. The digoxin‐labeled RNA probe (5′‐TTATCTGCGTAACTCTCCAC‐3′) was designed based on the back‐splicing junction of circEXOC7. After formaldehyde fixing and pepsin treating, we dehydrated cells in a gradient series of increasing concentrations of ethanol, incubated with specific RNA probes in hybridization buffer at 80°C for 2 min, then hybridized at 55°C for 2 h. The slides were washed, dehydrated, air‐dried, mounted with Prolong Gold Antifade Reagent with DAP, and visualized with LSM 800 confocal microscope (Carl Zeiss, Germany). CircEXOC7 was labeled by the RNA probe (red) and nuclei by DAPI (blue).

### 2.9 Chromatin immunoprecipitation assay

Protein A‐agarose and normal rabbit IgG were used to preclear cell lysates. Then AR antibody was added to the supernatant and incubated at 4°C for 12 h. The predicted sequence of the human DHX9 gene promoter was amplified using PCR reaction with specific primer sets and identified with agarose gel electrophoresis assay. IgG was chosen as a negative control. Primers' detailed information is shown in Table S1.

### 2.10 Luciferase reporter assay

The sequencing of DHX9 5′‐promoter region was amplified by PCR and inserted into the empty pGL3‐basic vector (Promega, Madison, WI, USA) for luciferase (Luc) reporter assay. The quick change mutagenesis method was used to achieve the site‐directed mutagenesis of the AR binding site. CSF1 3′UTR fragment (2039 bp) with mutant (MT) or wild type (WT) miRNA‐responsive elements were inserted into the psiCHECK‐2 vector (Promega). Following the manufacturer's protocol, cDNAs were transfected with the plasmids using Lipofectamine 3000 transfection reagent (Invitrogen). After 48 h incubation, the Luc activity was measured with Dual‐Luciferase Assay (Promega). PRL‐TK was considered as the baseline control response. All sequences’ information is shown in Table S1.

### 2.11 The circRNA‐miRNA pull‐down assay

Pre‐chilled lysis buffer supplemented with RNase inhibitor was used to lyse cells. Then Biotin‐labeled anti‐sense oligos (500 pM) against circEXOC7 (5′‐TTATCTGCGTAACTCTCCAC‐3′) was added into the cell lysate mixture and then rotated at 4°C for 12 h. 10 μl Streptavidin Agarose beads were mixed with the complex and rotated for 2 h at 4°C. After centrifugation, the beads were washed with pretreated cell lysis buffer five times. The total RNAs were extracted from the supernatant using Trizol reagent, and the pulled‐down miRNAs were detected with qRT‐PCR.

### 2.12 In vivo studies

Male 6 weeks old Balb/c nude mice were purchased from the Sipper BK Laboratory Animal Company (Shanghai, China) and divided into four groups (eight mice per group) for injection with Luc tranduced SW839 cells, which were pre‐cultured as follows: 1: vector control, 2: sh‐AR; 3: sh‐circEXOC7, and 4: sh‐AR+sh‐circEXOC7. Then 1 × 10^6^ of the prepared stable clones of SW839‐Luc cells suspended in 100 μL PBS were injected into the caudal artery of anesthetized mice using 29 G syringe needle in a short time (<3 s).^28^ Noninvasive In Vivo Fluorescent Imager (IVIS Spectrum, Caliper Life Sciences, USA) and X‐ray (Faxitron, Tucson, USA) were used to capture the images once a week. After 4 weeks, tumors as well as metastatic lesions were removed and counted for subsequent analysis.

### 2.13 Immunohistochemical staining

Animal tissue samples were fixed, embedded, and cut into 5 μm thick paraffin sections, affixed to slides, and allowed to dry over 12 h in the 37℃ incubator. After a series of processes, including dewaxing, rehydration, antigen retrieval, peroxidase quench, and blocking, specific primary antibodies (AR and CSF1) and biotinylated secondary antibodies were applied successively on the slides. After incubating, HRP‐streptavidin, DAB (Zymed, CA, USA) was used to detect HRP. The levels of specific antibodies were measured as the mean value of cell staining in three random fields at 400× magnification with Image‐pro Plus.

### 2.14 Alcian blue/orange G and TRAP+ staining

The detailed protocol has been attached in *Supplementary Materials*. Briefly, tissue paraffin slides were deparaffinized, hydrated to 70%, air dried, placed in acid‐alcohol for 30 s, then placed in Alcian Blue Hematoxylin for 25 min, washed gently in distilled water and differentiated in acid‐alcohol, and placed in 0.5% ammonium water and Eosin/Orange G successively. Then samples stained with Trap staining solution for 30 min at 37℃ with pink to red color representing osteoclasts, and pale blue to blue representing bone and cartilage.

### 2.15 Statistical analysis

SPSS 23.0 (SPSS Inc, CA, USA) and Graphpad Prism V6 (GraphPad Software, Inc., USA) were used to deal with all statistical analyses. Each phenotype assay was conducted in triplicate and at least three times. The data values were presented as the mean ± SD. For two independent groups, differences in mean values were analyzed by two‐tailed Student's *t*‐test. One‐way ANOVA followed by individual comparisons with Dunnett's test was used for more than two groups' comparison. Statistic results were considered significant when *p* ≤ 0.05.

## 3 RESULTS

### 3.1 The ccRCC has a gender difference and AR expression may be linked to the development of RBM

**FIGURE 1:**
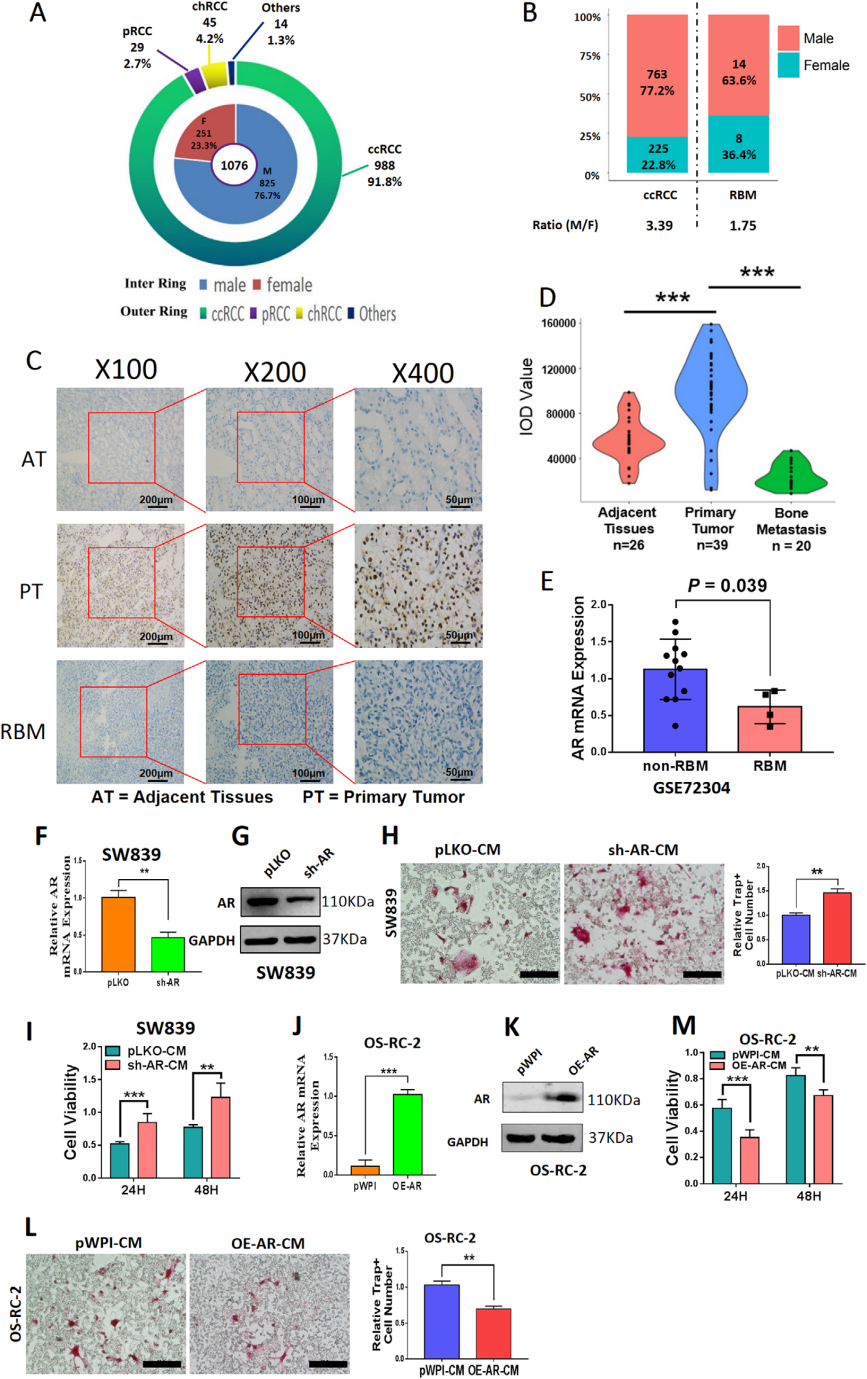
AR blunted BMMs osteoclastogenesis. (A and B) Gender ratio of RCC incidence in 1076 cases from Renji Hospital. (A) The inner ring chart shows the constituent ratio of male and female, and the outer ring chart shows the constituent ratio of tumor subtypes. (B) Gender differences within different groups of patients with ccRCC and RBM. (C) IHC staining showed that AR was mainly located in the nuclei. (D) The AR expression intensity of ccRCC in primary tumors (*n* = 39) was higher than that in adjacent tissues (*n* = 26) and RBM group (*n* = 20). (E) The mRNA expressions of AR in non‐metastatic ccRCC (*n* = 12), and RBM (*n* = 4) from GEO database (GSE72304). (F) Relative RNA levels of AR after knocking down AR (sh‐AR) in ccRCC SW839 cells. (G) Western blot assay was performed on SW839 cells with PLKO or sh‐AR to show AR protein levels. (H) Osteolytic formation assay was performed on BMMs to show sh‐AR‐CM from SW839 cells had increased osteoclast cell formation (scale bars, 200 μm). (I) BMMs were cultured in α‐MEM containing CM from SW839 cells transfected with sh‐AR (sh‐AR‐CM) or negative control (pLKO‐CM). (J) Relative RNA levels of AR after overexpressing AR (OE‐AR) in OS‐RC‐2 cells. (K) Western blot assay was performed to show AR protein level upon OE‐AR. (L) Osteolytic formation assay was performed on BMMs to show OE‐AR‐CM from OS‐RC‐2 cells had decreased osteoclast cell formation (scale bars, 200 μm). (M) BMMs were cultured in α‐MEM containing OE‐AR‐CM or negative control (pWPI‐CM) from OS‐RC‐2 cells. Surviving cells were counted at 24 h and 48 h. For (E‐F), (H), (J), (L‐M), data are presented as Mean ± SD, **p < 0.01, ***p < 0.001. Abbreviations: AT, adjacent tissues; chRCC, chromophobe renal cell carcinoma; pRCC, papillary renal cell carcinoma; PT, primary tumor; RBM, RCC bone metastasis

Our human clinical survey of 1076 RCC cases from Renji Hospital revealed that the male/female ratio is 3.29:1 (Figure 1A), and 91.8% of these RCC samples were ccRCC with the gender ratio at 3.39:1 (Figure 1B, Table S2).

Interestingly, among these human ccRCC samples, we found 22 ccRCC patients had bone metastases (RBMs) within 12 months after surgery, with the gender ratio at 1.75:1 (Figure 1B, Table S2). This significant gender difference suggests that sex hormones and related receptors may regulate RBM progression.

Our laboratory's long‐term focus is on AR, and we applied the RCC tissue array to examine the AR protein expression (Figure 1C). The results from AR staining revealed that AR expression was higher in the ccRCC primary tumors (*n* = 39) compared to the adjacent tissues (*n* = 26) and was significantly lower in RBM tissues (*n* = 20) than those found in the primary tumor group (*p* < 0.001) using Image‐Pro Plus software (Figure 1D, Table S3). These human clinical sample data were also confirmed via analysis from GEO database (GSE72304), showing the AR mRNA expressions in the non‐RBM group (*n* = 12) are much higher as compared to those from the RBM group (*n* = 4, *p* = 0.039) (Figure 1E).

Based on the results of Figures 1A‐1E and Tables S2‐3, we confirmed that ccRCC has a gender difference, and higher AR expression may be linked to fewer RBMs.

### 3.2 AR expression in the ccRCC cells is inversely correlated with RBM and osteolytic formation of BMMs cells

To verify the above human clinical survey data showing AR expression may be negatively linked to RBM, as early studies indicated that increased osteolytic formation can be seen in local bone tissue during the process of bone metastasis,^9^, ^29^ we focused on whether AR could impact the osteolytic formation. Our early studies demonstrated that AR expressed differently in different RCC cells, being higher in SW839 cells and lower in OS‐RC‐2 and 786‐O cells.^11^

We first downregulated AR in SW839 cells (Figures 1F and 1G) and collected the CM to study their potential impact on the primary BMMs and the osteolytic formation. The results revealed that BMMs treated with CM from AR‐downregulated SW839 cells led to more differentiation to TRAP‐positive multinucleated osteoclasts (Figures 1H and S1A) with higher proliferation (Figure 1I) at both 24 and 48 h. In contrast, the BMMs treated with the CM from AR‐upregulated OS‐RC‐2 cells (Figures 1J and 1K) displayed less osteolytic formation (Figures 1L and S1B) and lower proliferation (Figure 1M).

Results from Figures 1F‐1M indicated that AR may decrease the osteolytic formation of BMMs cells, which is negatively associated with RBM progression.

### 3.3 AR decreased osteolytic formation by altering the circRNA expression

Published studies demonstrated that non‐coding RNAs played a significant role in osteoblastic/osteolytic conversion.^30^, ^31^ To explore the mechanism, we focused on the downstream circRNAs expression, which plays significant roles in tumor progression,^32^ including RCC metastasis.^27^

**FIGURE 2:**
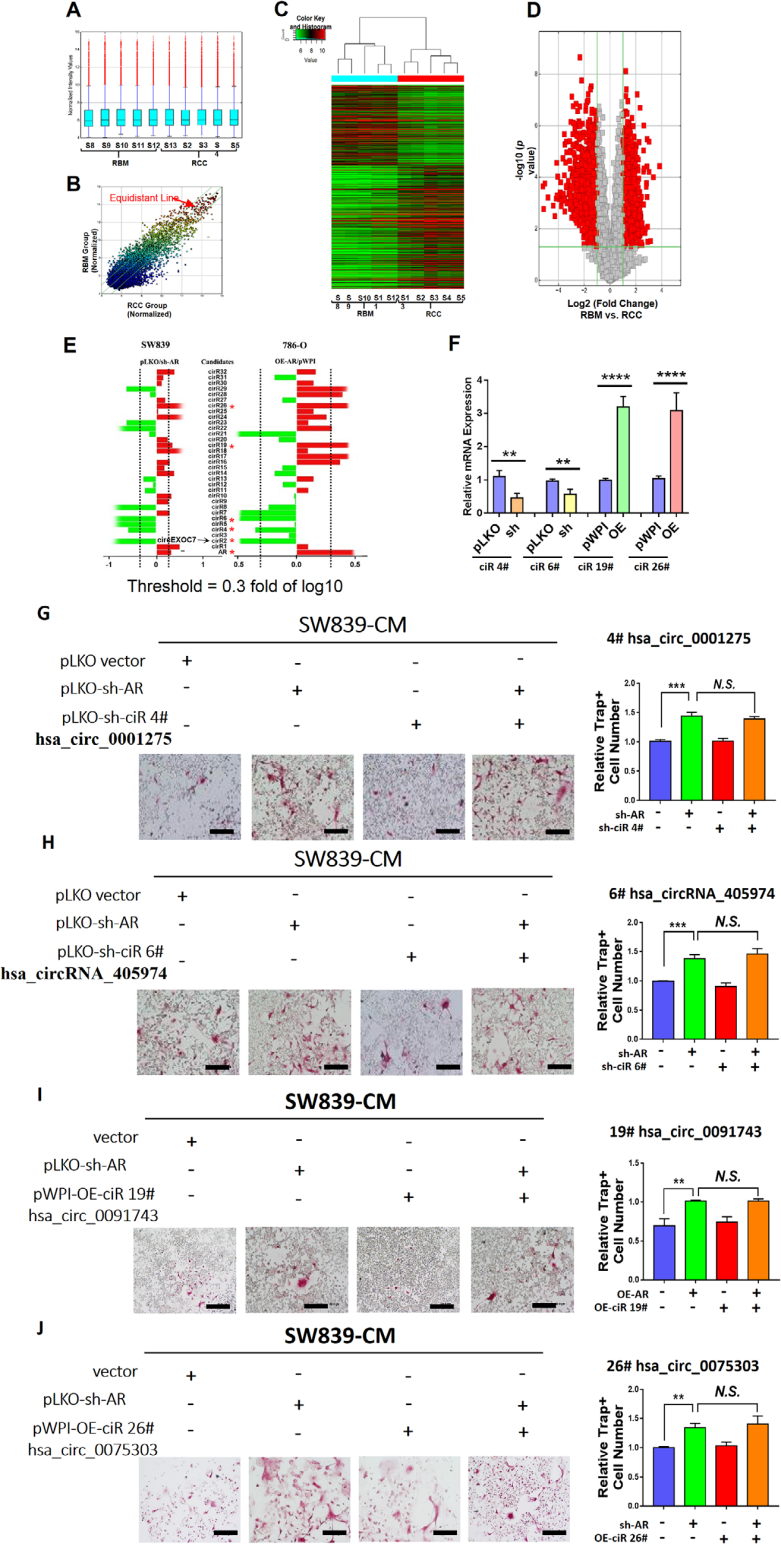
CircRNA microarray and screening process. Results from circRNA microarray and bioinformatics analysis from five paired human RCC vs. RBM tissues were summarized in (A‐D). (A) Box plot demonstrated no significant differences in the distribution of intensity across 10 clinical samples. (B) Scatter plot displayed circRNAs expression correlation between RCC and RBM group. (C) Heat map generated from circRNAs microarray data reflecting circRNAs expression values in RCC and RBM groups. (D) Volcano plot showing DERs data. The red points indicate points‐of‐interest that display both large magnitude fold‐changes (x‐axis) and high statistical significance (‐log10 of *p*‐value, y‐axis). The green line shows points above the line having *p *< 0.05 and points below the line having *p *> 0.05. This plot is colored such that those points having a fold‐change less than 2 (log2 = 1) are shown in gray. (E) The qRT‐PCR was performed to show 32 selected candidate circRNAs expression levels in cells. (F) The qRT‐PCR was performed on SW839 cells to show effects of manipulating efficiency of selected candidate circRNAs, ciR4#, ciR6#, ciR19#, and ciR26#. (G‐J) Interruption assays revealed that suppressing (G) ciR‐4# (hsa_circ_0001275), (H) ciR‐6# (hsa_circRNA_405974), expression cannot reverse/block the knocked‐down AR‐increased osteolytic formation in SW839 cells (scale bars, 200 μm), increasing (I) ciR‐19# (hsa_circ_0091743) expression, and (J) increasing ciR‐26# hsa_circ_0075303 expression cannot reverse/block the AR‐suppressed osteolytic formation in SW839 cell line (scale bars, 200 μm). For (E‐J), quantitation is at the right, and data are presented as Mean ± SD, **p < 0.01, ***p < 0.001, ****p < 0.0001. Abbreviation: N.S., not significant

We first applied the Arraystar human circRNA V2.0 microarray and Bioinformatics analysis to compare the difference between five unpaired human RCC vs. RBM tissues (Figures 2A‐2D). There was no significant difference in the distribution of intensity across the 10 clinical samples (Figure 2A). Scatter plot (Figure 2B), heat map (Figure 2C), and volcano plot (Figure 2D) demonstrated the differential expression of circRNAs (DERs). We then selected the top 32 DERs (16 upregulated and 16 downregulated DERs) from the summarized results for further confirmation using the qRT‐PCR assay. The results demonstrated that two circRNAs were upregulated (cirR26, and cirR19) and three circRNAs (cirR6, cirR4, and cirR2) were downregulated in ccRCC cell lines in response to altered AR expression (upregulated AR in 786‐O cells and downregulated AR in SW839 cells) by using circRNA‐specific divergent primers (Figure 2E and Table S4).

Among the five AR‐altered circRNAs, four of them failed to reverse the AR's effect on osteolytic formation in BMMs cells (Figures 2F‐2J).

Results from Figures 2A‐2J reveal that AR may function by regulating some selective circRNAs that are differentially expressed in the ccRCC vs. RBM to affect the osteolytic formation.

### 3.4 AR suppressed osteolytic formation by decreasing the circEXOC7 expression

We then focused our study on the circRNA_0092355 and renamed it circEXOC7 as it is derived from the host gene EXOC7. Early studies indicated that alternative splicing of EXOC7 could regulate the inflammatory secretome and the pro‐tumorigenic effects,^33^ while alternative splicing is likely a significant contributor to circRNA formation.

**FIGURE 3:**
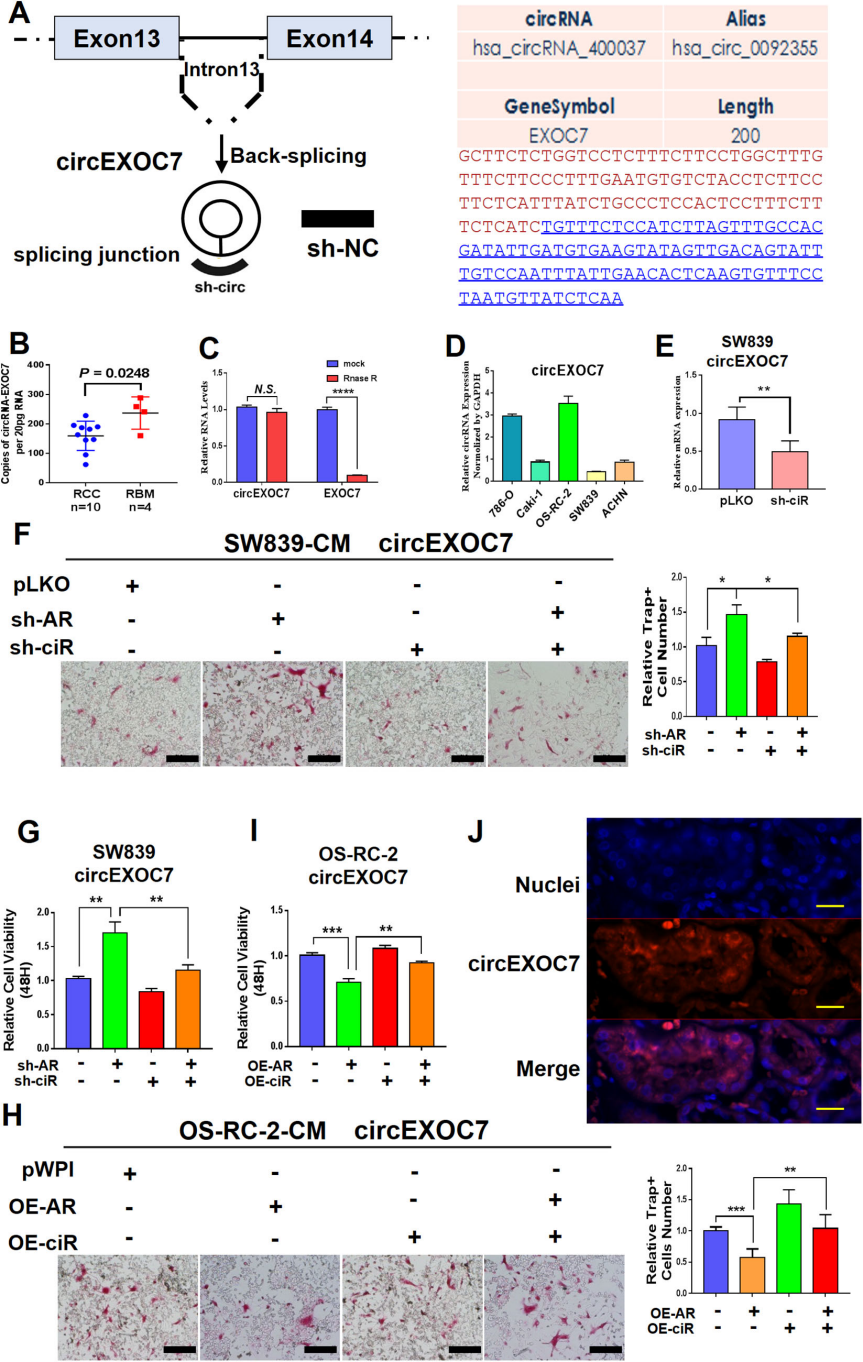
The characteristics of circEXOC7. (A) The circEXOC7 information in circBase database (http://www.circbase.org/). (B) The qRT‐PCR was performed to show quantification of circEXOC7 in RCC (*n* = 10) and RBM (*n* = 4) tissues (*p* = 0.0248). (C) RNase R assay showed no significant changes in circEXOC7 after RNase R treatment. (D and E) The qRT‐PCR was performed to show (D) circEXOC7 expression in different RCC cell lines and (E) to show knockdown efficiency of selected candidate circEXOC7. (F and G) The interruption approaches revealed that suppressing circEXOC7 expression led to (F) partially reverse knocked‐down AR‐increased osteolytic formation in SW839 cells, led to (G) partially reverse knocked‐down AR‐increased proliferation in SW839 cells (scale bars, 200 μm). (H and I) Increasing circEXOC7 expression also led to partially reverse OE‐AR‐suppressed (H) osteolytic formation in OS‐RC‐2 cells and (I) proliferation in OS‐RC‐2 cells (scale bars, 200 μm). (J) RNA‐FISH assay in RCC tumor tissues revealed that higher circEXOC7 expression was detected in cytosol as compared to those found in nuclei in human clinical ccRCC samples (nuclei were stained with DAPI. Scale bar, 100 μm). For F and H, quantitation is at the right, for (B‐I), data are presented as Mean ± SD, *p < 0.05, **p < 0.01, ***p < 0.001, ****p < 0.0001. Abbreviation: N.S., not significant

The circEXOC7 is generated from the sequence of EXOC7 (Figure 3A). We first studied its potential significance in a clinical sample survey, and the results showed that circEXOC7 expression was much higher in patients with RBM (*n* = 4) than those found in RCC patients without RBM (*n* = 10) (*p* = 0.0248) (Figure 3B), which is in agreement with results from the circRNA array assay. Importantly, the results from the RNase R assay also showed no significant changes in circEXOC7 compared to EXOC7 (Figure 3C), suggesting circEXOC7 is a true circular RNA, but not a linear RNA.

We also tested the circEXOC7 expression in different RCC cell lines (Figure 3D) and then constructed the shRNAs for circEXOC7 (Figure 3E), and results from the interruption approaches revealed that suppressing circEXOC7 expression led to partially reverse AR's effect on regulating the osteolytic formation of BMM cells (Figure 3F) and proliferation (Figure 3G) of SW839 cells. In contrast, increasing circEXOC7 expression also led to partially blocking the effect that downregulated AR promotes osteolytic formation of BMM cells (Figure 3H) and proliferation (Figure 3I) in OS‐RC‐2 cells. In addition, RNA‐FISH assay revealed that higher circEXOC7 expression was detected in cytoplasm as compared with the nucleus in human clinical ccRCC samples (Figure 3J).

Results from Figures 3A‐3J demonstrate that AR suppressed osteolytic formation via decreasing circEXOC7 expression.

### 3.5 AR altered circEXOC7 expression via transcriptional regulation of DHX9 expression

**FIGURE 4:**
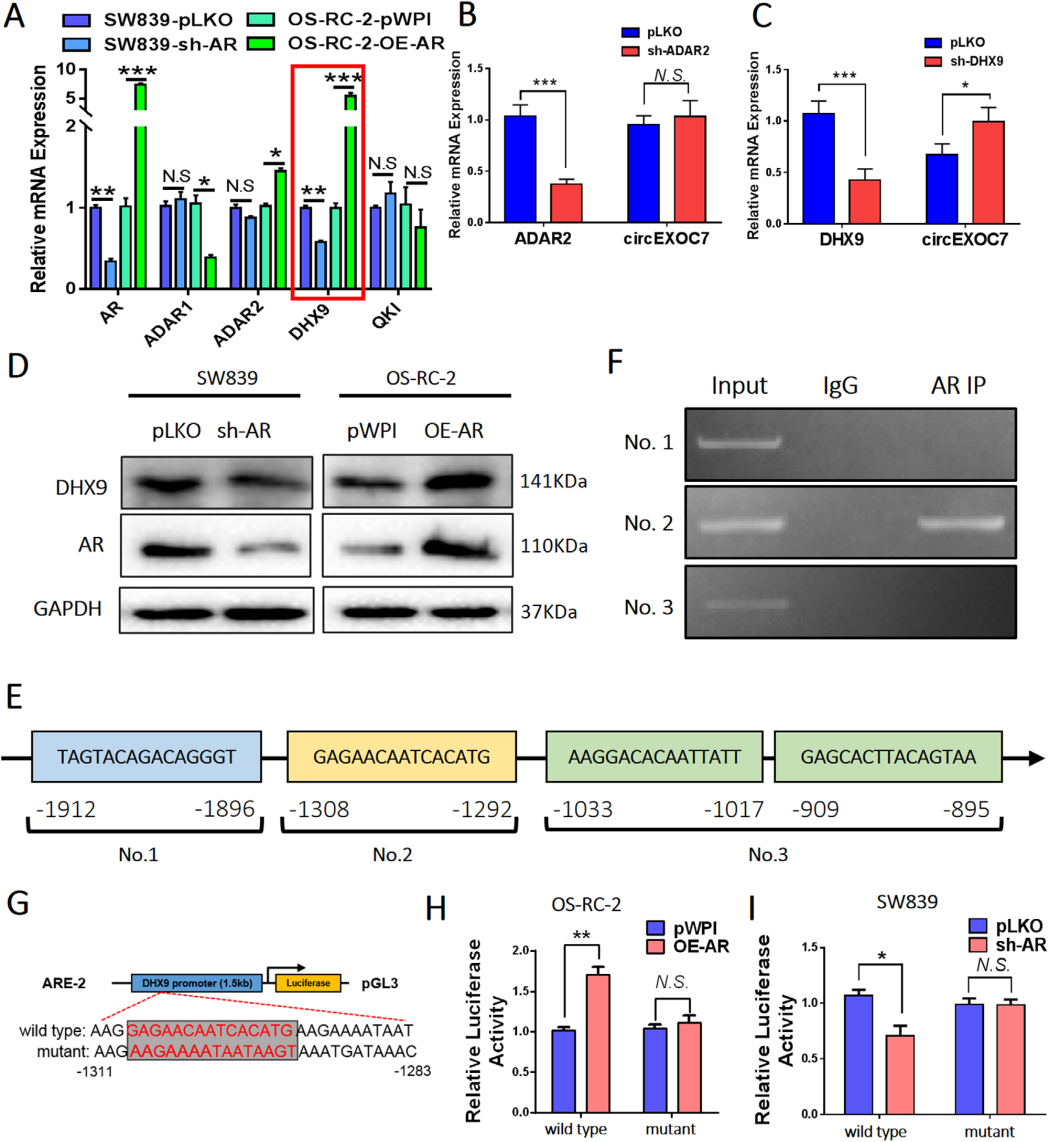
The expression of circEXOC7 is regulated by DHX9. (A) The qRT‐PCR was performed on SW839 and OS‐RC‐2 cell lines to show correlation between AR and four genes related to circRNA biogenesis. Among them, ADAR2 and DHX9 have a positive correlation with AR mRNA expression. (B and C) The qRT‐qPCR was performed to show (B) knocking down DHX9, and not (C) ADAR2 gene, led to increased circEXOC7 expression in SW839 cells. (D) WB was performed on SW839 and OS‐RC‐2 cells to show that modulated AR expression also altered DHX9 expression. (E) Predicted AR binding sites on the DHX9 promoter region using website http://jaspar.genereg.net/. (F) ChIP assay showing AR could bind to the No. 2 potential ARE on the DHX9 5′‐promoter region. (G) The wild type and mutant pGL3‐DHX9 promoter reporter constructs. (H and I) Luciferase activity after transfection of wild type or mutant DHX9 promoter reporter constructs in OS‐RC‐2 cells (H) transfected with AR‐cDNA or pWPI and SW839 cells (I) transfected with AR‐shRNA or pLKO. For (B and C), (H and I), data are presented as Mean ± SD, *p < 0.05, **p < 0.01, ***p < 0.001. Abbreviation: N.S., not significant

To examine how AR regulates circEXOC7, we tested, but failed to find a significant change of expression of its host gene EXOC7 under the influence of AR. Previous reports confirmed that circRNA synthesis can be regulated at the post‐transcriptional level through RNA‐binding proteins critical for circRNA production.^21^, ^34^, ^35^, ^36^ Thus, we tested the impact of AR expression on the four genes, ADAR1, ADAR2, DHX9, and QKI, and the results revealed that ADAR2 and DHX9 expressions have a positive correlation with AR mRNA expression (Figure 4A). However, only knocking down DHX9, and not ADAR2 gene, could increase the circEXOC7 expression in SW839 cells (Figures 4B and 4C). Importantly, AR alteration also led to modulate the DHX9 expression in both SW839 and OS‐RC‐2 cells (Figure 4D), suggesting AR may be able to regulate the circEXOC7 expression via modulating the DHX9 expression.

To further explore the specific molecular mechanism, we applied the bioinformatics analysis on the website (http://jaspar.genereg.net/) to identify the potential androgen response elements (AREs) on the DHX9 5′‐promoter, and results revealed four AREs within this region (Figure 4E). Results from chromatin immunoprecipitation assay with the AR antibody showed AR could bind to the potential ARE No.2 (‐1308 to ‐1292) on the DHX9 promoter region (Figure 4F). We then constructed the wild type and mutant pGL3‐DHX9 promoter reporter plasmids (Figure 4G), and results from Luc report assay demonstrated that transfecting with AR‐cDNA in wild type OS‐RC‐2 cells led to increasing the Luc activity, and transfecting with sh‐AR in wild type SW839 cells led to decreasing the Luc activity. This phenomenon cannot be observed in the mutant transfected cells (Figures 4H and 4I).

Together, AR can transcriptionally induce DHX9 expression by directly binding to the ARE No.2 on the DHX9 gene promoter to alter circEXOC7 expression in ccRCC cells.

### 3.6 AR/DHX9/circEXOC7 axis could alter osteolytic formation by regulating the CSF1 expression

**FIGURE 5:**
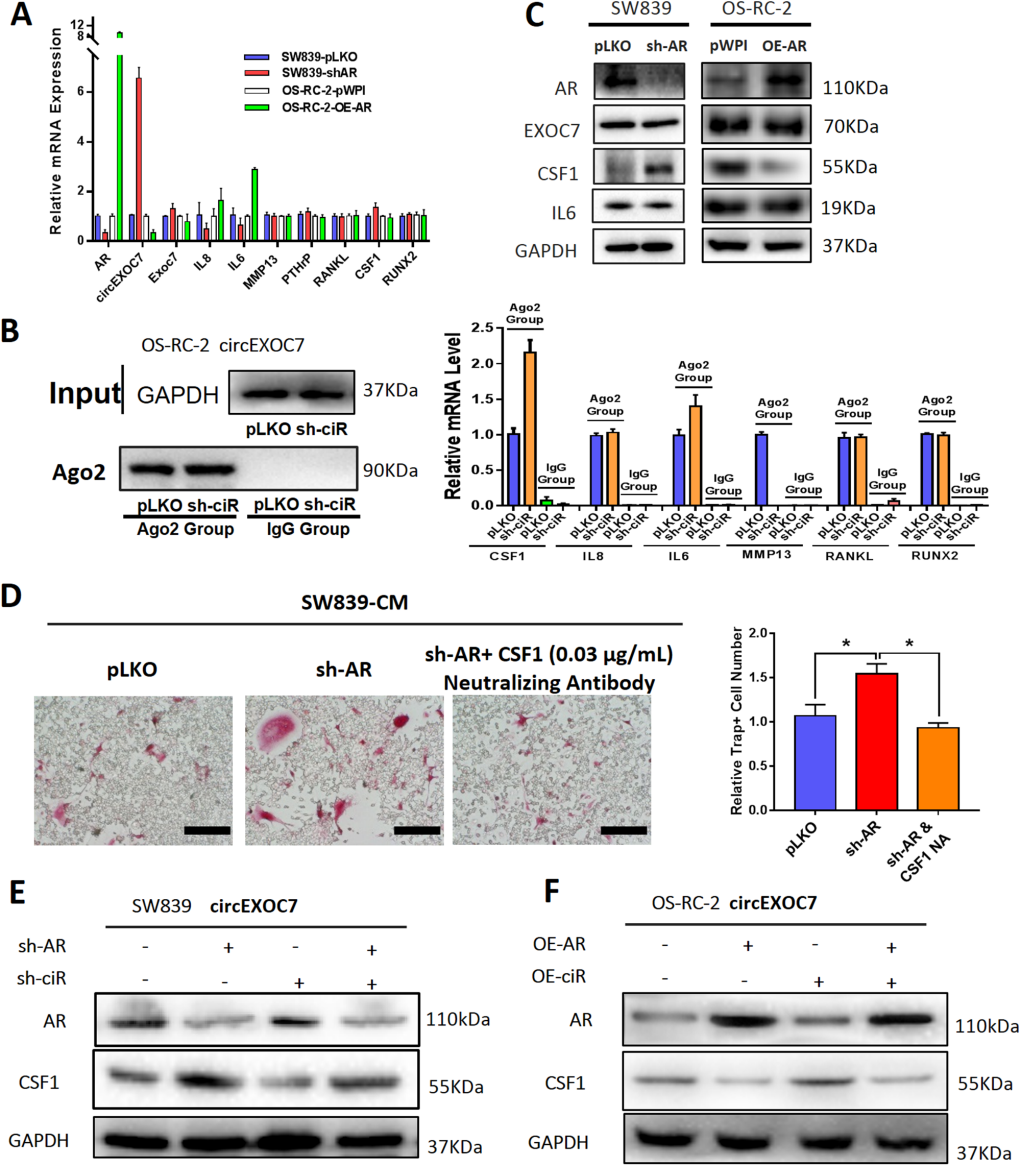
AR/DHX9/circEXOC7 axis could alter osteolytic formation by regulating the CSF1 expression. (A) The qRT‐PCR analysis revealed that IL8 and IL6 have correlated changes in response to AR in SW839 and OS‐RC‐2 cell lines; others have no obvious change in mRNA levels. (B) Ago2 RIP assay to detect the CSF1 mRNA in Argonaute 2 (Ago2) complex and results revealed that knocking‐down circEXOC7 (sh‐ciR) increased CSF1 mRNA level. (C) WB revealed that CSF1 was significantly increased after knocking down AR in SW839 (sh‐AR) and decreased after overexpressing AR (OE‐AR) in OS‐RC‐2 cell lines. (D) The interruption approach using CSF1 neutralizing antibody or CSF1‐shRNA in SW839 partially reversed sh‐AR‐altered osteolytic formation (scale bars, 200 μm, and quantification at the right). (E) The interruption approaches revealed that knocking down circEXOC7 could reverse sh‐AR's negative effect on CSF1 protein expression in SW839 cells. (F) The impact of OE‐AR on the CSF1 protein level could also be partially blocked by up‐regulating circEXOC7 in OS‐RC‐2 cell line. For (E), data are presented as Mean ± SD, *p < 0.05

Next, we focused on the osteolytic formation‐related genes: IL8, IL6, MMP13, PTHrP, RANKL, CSF1, and RUNX2.^29^, ^37^ Results from the qRT‐PCR demonstrated that IL6 mRNA expression, not others, was significantly altered after changes of AR expression in SW839 and OS‐RC‐2 cell lines (Figure 5A). As circEXOC7 is involved in this regulation, miRNAs might likely participate in this process since circEXOC7 was mainly located in the cell cytoplasm. To directly test this possibility, we implemented immunoprecipitation of Argonaute 2 (Ago2) followed by RNA detection (RNA‐immunoprecipitation, Ago2 RIP) and tested the candidate mRNAs that can be potentially regulated by miRNA. The results showed that CSF1 could be detected more in the Ago2 RIP assay in cells with knocked‐down circEXOC7 (Figure 5B), suggesting that CSF1 might be a candidate mRNA to regulate osteolytic formation by circEXOC7. Indeed, western blot analysis revealed that CSF1 was significantly increased after knocking‐down AR in SW839 cells and decreased after over‐expressing AR in OS‐RC‐2 cells (Figure 5C). Although IL6 mRNA was altered in response to AR expression, there is little change in protein expression and was not investigated further in this study. In contrast, we found the interruption approaches using CSF1 neutralizing antibody or CSF1‐shRNA in SW839 cells all led to partially reverse the AR‐altered osteolytic formation (Figure 5D). Also, results from the interruption approaches confirmed that knocking‐down circEXOC7 could partially reverse AR‐suppressed CSF1 protein expression in SW839 cells (Figure 5E), and increasing circEXOC7 expression could partially reverse/block the increased‐AR effect on CSF1 expression in OS‐RC‐2 cells (Figure 5F).

Figures 5A‐5F suggests that the AR/DHX9/circEXO7 axis may function through regulating CSF1 expression to alter the osteolytic formation.

### 3.7 circEXOC7 alters CSF1 expression via sponging and suppressing miR‐149‐3p

Our findings that AR/circEXOC7 can alter CSF1 function, with little impact on the CSF1 mRNA expression, led to a logical hypothesis that CSF1 may be regulated by AR through miRNAs that can bind to circEXOC7. In addition, the Ago2 RIP assay provided evidence that miRNAs play a pivotal role in this process.

**FIGURE 6:**
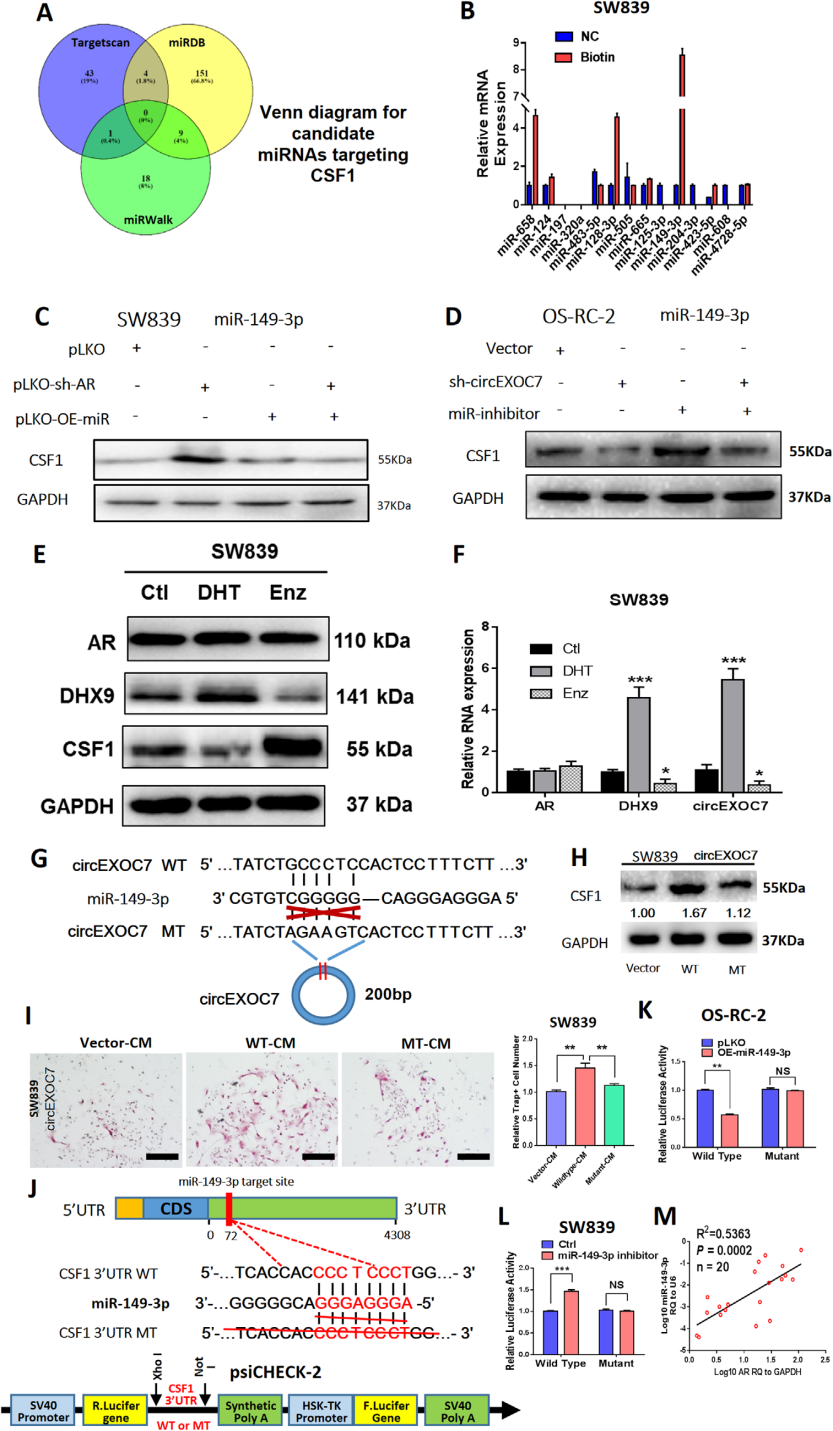
Mechanism of circEXOC7 regulation of CSF1 in ccRCC. (A) Venn diagram for prediction of CSF1 potential miRNAs using three miRNA‐related websites (Targetscan, miRDB, and miRWalk). (B) Pull‐down assay demonstrated potential direct binding of three candidate miRNAs with circEXOC7. (C) WB for miRNA rescue assay revealed that suppressing AR led to an increase in CSF1 expression, which could then be partially reversed/blocked via adding miR149‐3p in SW839 cells. (D) WB for miRNA rescue assay showed that circEXOC7 decreased CSF1 expression, which could also be partially reversed/blocked by miR149‐3p inhibitor in OS‐RC‐2 cells. (E) AR, DHX9 and CSF1 expressions by WB analysis in SW839 cells treated with DHT and Enz treatment. (F) The mRNA levels of DHX9 and circEXOC7 expression by qPCR assay in SW839 cells treated with DHT and Enz treatment. (G) The circEXOC7 with and without miR‐149‐3p binding sites (CircNet). (H) WB was performed to show MT circEXOC7 failed to significantly increase CSF1 expression in SW839 cells. (I) Osteolytic formation assay showed that MT circEXOC7 could not increase osteoclast cell formation (scale bars, 200 μm). (J) Sequence alignment of CSF1 3′UTR with WT and potential MT miR‐149‐3p targeting sites. The lower panel shows the psiCHECK‐2 vector information, restriction enzyme cutting sites, and insertion site. (K and L) Luciferase reporter activity after transfection of wild type or mutant CSF1 3′UTR reporter constructs in OS‐RC‐2 cells with/without OE‐miR‐149‐3p and SW839 cells treated with/without miR‐149‐3p inhibitor compared to the control cells. (M) Association of AR expression with miR‐149‐3p expression in ccRCC tumors analyzed by linear regression (*p* = 0.0002, R^2 ^= 0.5363, *n* = 20). For (I) quantitation is at the right, for (F), (I), (K and L), data are presented as Mean ± SD, *p < 0.05, **p < 0.01, ***p < 0.001. Abbreviation: N.S., not significant

To identify potential miRNAs that can be sponged by circEXOC7 and also bind to the CSF1 3′UTR, we selected candidates from online databases (miRDB, TargetScan, and miRWalk) and identified 14 potential candidates (Figure 6A). RNA pull‐down assay was performed using the biotinylated oligonucleotides (5′‐AGAGAAGCTTGAGATAACATT‐3′) to target the circular back‐splicing junction to test whether circEXCO7 could interact with these candidates. We found that miR‐658, miR‐128‐3p, and miR‐149‐3p were enriched, suggesting potential direct binding of these three candidates' miRNAs with circEXOC7 (Figure 6B).

The rescue assays proved that suppressing AR led to increasing CSF1 expression in SW839 cells, which could be partially reversed by miR149‐3p (Figure 6C). Similarly, the downregulated circEXOC7 (sh‐circEXOC7) decreased CSF1 expression in OS‐RC‐2 cells, which could also be partially reversed by transducing miR149‐3p inhibitor (Figure 6D). However, the other two candidates, miR‐658 and miR‐128‐3p, failed to block the knocked‐down AR‐increased CSF1 protein expression (Figure S2).

To confirm these results, RCC cells were treated with the androgen dihydrotestosterone (DHT), vehicle, or the anti‐androgen enzalutamide (Enz). The western blot results showed that DHX9 was increased by DHT and decreased by Enz in SW839 cells. However, CSF1 decreased with DHT treatment and increased with Enz treatment (Figure 6E). The quantitative PCR assay confirmed that the mRNA levels of DHX9 and circEXOC7 expressions also significantly increased by DHT treatment and decreased by Enz treatment (Figure 6F).

This indicated that the circEXOC7 may function via sponging miR‐149‐3p to regulate CSF1 expression to impact the osteolytic formation.

### 3.8 The miR‐149‐3p directly binds to the CSF1 3′UTR region

The presumptive binding site of miR‐149‐3p was predicted with the bioinformatics method (using database *CircNet*). We constructed the MT circEXOC7 (Figure 6G) with the mutant miRNA binding site. As expected, WT circEXOC7, but not MT circEXOC7, had significantly increased CSF1 expression (Figure 6H) and osteolytic formation in SW839 cells (Figure 6I), suggesting that circEXOC7 could enhance CSF1 expression/function via sponging miR‐149‐3p.

Next, we identified presumptive binding sites located on the 3′UTR of CSF1 mRNA (using database *miRDB*) with subsequent construction of the reporter plasmids with the psiCHECK‐2 vector carrying the WT or MT miRNA‐target sites (Figure 6J). The Luc reporter assay demonstrated that miR‐149‐3p significantly decreased Luc activity in OS‐RC‐2 cells transfected with WT CSF1 3′UTR, but not the MT, CSF1 3′UTR (Figure 6K) and attenuating miR‐141‐3p markedly increased Luc activity in SW839 cells, but not the MT CSF1 3′UTR (Figure 6L). A clinical survey of ccRCC AR‐positive tumors also proved that the miR‐149‐3p and AR have a positive correlation (Figure 6M).

### 3.9 Animal model verification of AR/DHX9/circEXOC7/miR‐149‐3p/CSF1 signaling

To test the validity of this signaling pathway in vivo, we applied the preclinical study using intra‐caudal arterial implantation of the SW839 cells expressing firefly Luc and transfected with vector control, AR‐shRNA, and circEXOC7‐shRNA. The mice were divided into four groups and injected with vector control (Group 1), AR‐shRNA (Group 2), circEXOC7‐shRNA R‐149‐3p/CSF1 signaling (Group 3), or AR‐shRNA and circEXOC7‐shRNA (Group 4).

**FIGURE 7:**
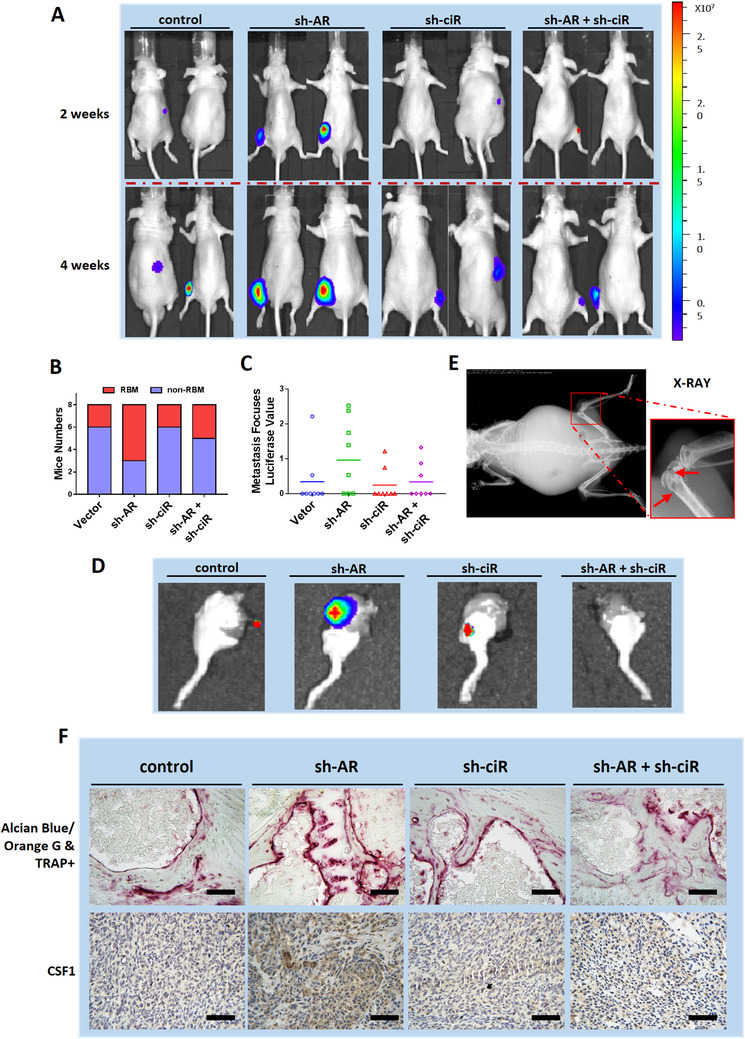
Animal model verification of the AR/DHX9/circEXOC7/miR‐149‐3p/CSF1 signaling in RBM. (A) IVIS imaging exhibited the RBM sizes in mice of various groups. (B) Quantitative analysis of RBM mice numbers in each group. (C) Quantitative analysis of luciferase values of tumors in each group. (D) Tumor tissues were obtained for imaging after sacrificing the mice. (E) Small animal X‐ray studies were carried out to prove RBM‐related osteolytic bone tissue damage. (F) Alcian Blue/Orange G and TRAP+ stainings were performed to confirm osteoclasts formation and bone tissue homeostasis. IHC staining was performed to evaluate CSF1 expression on mice bone metastasis foci. (Scale bars, 200 μm)

After 4 weeks since injection, the mice were sacrificed, and the metastatic sites were examined.^38^ The results revealed that the group that received AR‐shRNA injection clearly developed more and larger RBM than the control group. In addition, circEXOC7‐shRNA could suppress RBM development (Figures 7A‐7D). X‐ray studies were carried out and also confirmed that the RBM could cause osteolytic damage (Figure 7E).

Alcian Blue/Orange G and TRAP+ staining also demonstrated that the expression of osteoclasts was higher, and bone tissue damage was more obvious in the sh‐AR group compared to the vehicle control. We also found that sh‐circEXOC7 could partially reverse those effects (Figure 7F, upper panel). Immunohistochemical staining staining also verified that CSF1 expression was higher in sh‐AR group compared to the control group, and using sh‐circEXOC7 led to suppress the sh‐AR increased CSF1 expression (Figure 7F, lower panel).

These animal study results from the in vivo intra‐caudal arterial injection mouse model (Figures 7A‐7F) were consistent with in vitro cell lines data showing targeting the AR and circEXOC7 could lead to altering RBM developm ent via altering the AR/DHX9/circEXOC7/miR‐149‐3p/CSF1 signaling pathway.

## 4 DISCUSSION

**FIGURE 8:**
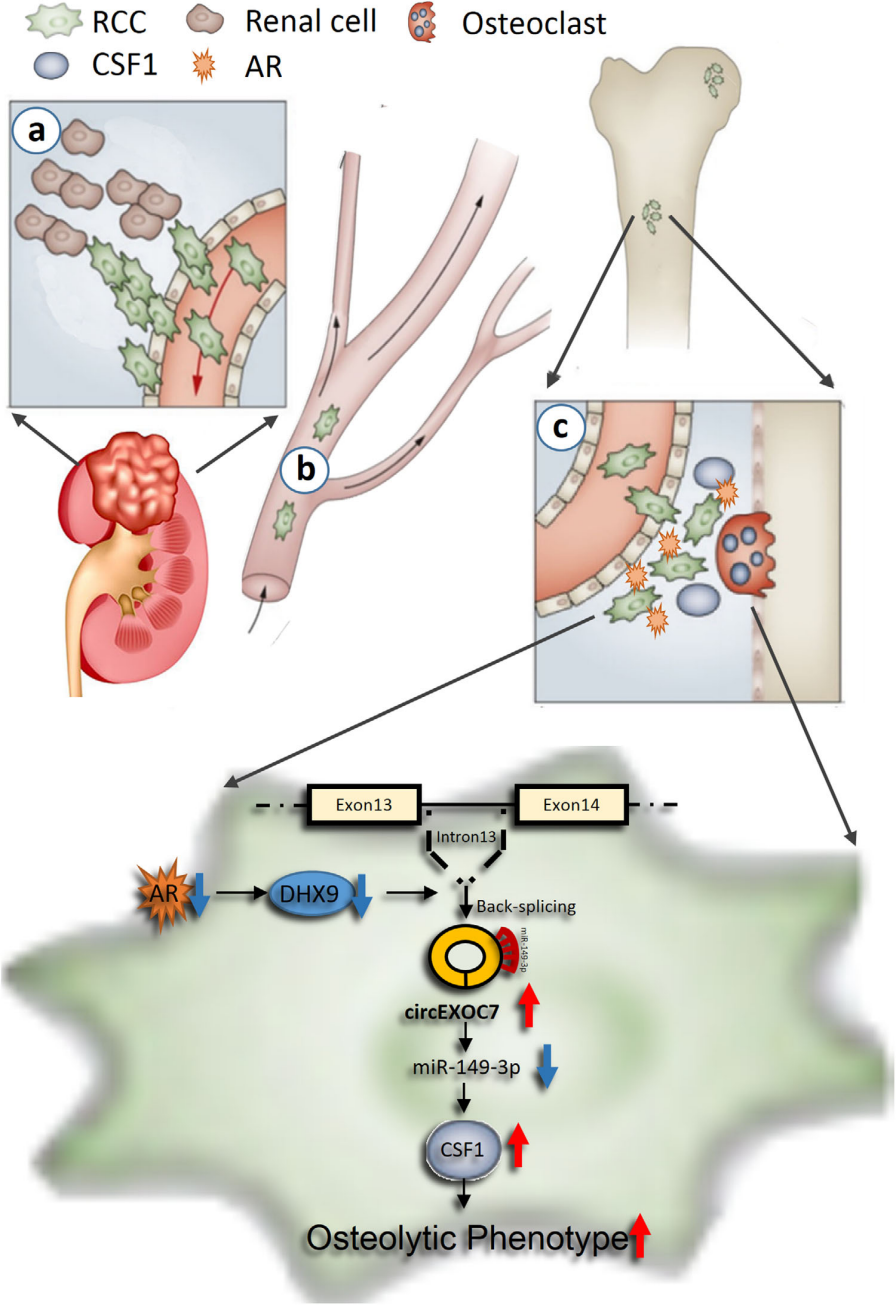
Schematic illustration of AR's role in RBM progression. a, extravasation; b, circulation; c, intravasation and colonization

RBM is a multistage process that may involve at least three major steps from migration/invasion of the RCC cells to distant bone tumor formation via lymphatic and vascular circulatory systems (Figure 8).^39^ It is likely that a subset of RCC cells initially acquires the ability to escape from the tumor in the kidney. The 2nd step involves the blood vessel intravasation with the capability to survive in the circulation and then extravasation from the blood vessels. The 3rd step involves colonization competence and tumor growth in the bone microenvironment. The success of these three steps can then lead to the development of more aggressive metastatic tumors in the bone tissues.^9^, ^39^, ^40^

Our current studies focus on the role of AR in RCC during the 3rd step that involves altering the osteolytic formation to impact the RBM. The bone microenvironment contains a mineralized extracellular matrix and several specific cell types to form a fertile soil for many tumors to grow and expand.^37^ Several solid tumors can metastasize to the bone and induce osteolytic and/or osteoblastic lesions through secreting certain key proteins to impact the balance of osteoclasts and osteoblasts formation.^41^, ^42^ The consequences of these changes may then lead to an increase in the tumor colonization in the bone.^43^, ^44^ So far, several tumor‐derived osteoclast‐activating factors were identified, including RANKL, CSF1, IL‐6, IL34, MMP2, MMP13, RUNX2, and PTHrP.^37^, ^42^

Here we found ccRCC AR may function through regulating CSF1 expression to impact the osteolytic formation to influence the RBM progression. Further mechanism dissection revealed that AR might regulate CSF1 expression by altering the DHX9/circEXOC7/miR‐149‐3p/CSF1 signaling. This is consistent with early studies showing non‐coding RNAs, including circRNAs, can play important roles to influence tumor progression.^45^, ^46^

Our study reconciled AR low expression in some RCC tissues as described in the previous report^14^ as these limited number of tissues were enriched with lymph node^15^ or bone metastases, reflecting the impact of AR expression in RCC progression as outlined in our current research.

The current studies expanded our earlier findings on the role of AR in regulating RCC metastasis. In addition to the bone, the other two common metastatic sites for RCC are lung and lymph nodes.^7^ AR can regulate VEGF‐A expression, thereby affecting RCC angiogenesis and eventual lung metastasis. On the other hand, AR regulation of VEGF‐C expression can affect RCC lymphangiogenesis and lymphatic metastasis.^15^

Our studies also substantiate early findings that non‐coding RNAs play a crucial role in the initiation and progression of multiple cancers.^46^, ^47^, ^48^, ^49^, ^50^ Compared to their linear counterparts, the circRNAs are widely and highly expressed in a variety of human cells^51^ and are less prone to be degraded by RNA exo‐enzymes due to their unique closed‐loop structure^52^ and may function by regulating the oncogenic or suppressor genes to intervene with tumor progressions.^20^, ^53^ Previous studies demonstrated that DHX9 inhibits the synthesis of circRNAs through binding to their flanking inverted complementary sequences and inhibiting the pairing of these sequences.^54^ We found that circEXOC7 appears to be regulated by this mechanism.

Previously, several studies proved that miR‐149‐3p plays a very important role as a regulator of cell cycle progression and apoptosis.^55^ In our research, we demonstrated that miR‐149‐3p was downregulated in RBM and reduced through sponging by circEXOC7, thus resulting in increased CSF1 expression and osteolytic formation.

Our clinical survey demonstrated the gender ratio (male/female) in ccRCC is 3.39:1 and in RBM is 1.75:1. A previous study also indicated that the ccRCC patients' gender difference of male to female is 2.8:1, the ratio of ccRCC lung metastasis patients is 4.9:1, while in lymphatic metastases is 1.7:1.^15^ These differing ratios likely reflect different collection sources from individual hospitals, nevertheless, the trend is consistent with a clear gender difference in RCC formation and metastasis in that there are more male RCC patients, yet their metastases are more to the lung than to the bone and lymph nodes. These differential metastatic locations are likely influenced by sex hormones and their receptors as illustrated in this report. This clear demonstration of gender and AR's role in our studies is contrasted by a finding from the analysis of a TCGA dataset that the AR level has no difference between male (6.628 ± 0.09701, *n* = 345) and female (6.777 ± 0.1286, *n* = 188) (*p* = 0.3571) tumors using the KIRC dataset (*n* = 533) (Figure S3). Several possibilities could explain this difference. Serum androgen levels between males and females may be one of the most critical issues. At the same time, a lack of difference in AR expression in males and females in principle supports the role of AR as required in RCC initiation and development. On the other hand, this TCGA data represented the mRNA level, thus there are many layers of regulation toward final protein abundance and function, and mRNA levels do not necessarily represent the final functional states of the encoded proteins. Also, as AR is a nuclear hormone receptor, the difference in ligand bioactivity and the ultimate AR function might be regulated in a manner dependent upon tumor stage, tumor microenvironment, as well as a potential expression of its target molecules.

## 5 CONCLUSION

Our study identified the AR/circEXOC7miR143‐3p/CSF1 signaling pathway (Figure 8), which is associated with ccRCC osteolytic formation and bone metastasis. Preclinical studies demonstrated that targeting circEXOC7 can be a novel promising approach to overcome RBM progression, establishing the foundation for further developing related small molecular inhibitors to slow‐down/block the RBM progression in patients.

## CONFLICT OF INTEREST

The authors declare no conflict of interest.

## ETHICS APPROVAL AND CONSENT TO PARTICIPATE

The Ethical Committee and Institutional Review Board of the Renji Hospital Shanghai Jiao Tong University reviewed and approved this study protocol. All patients signed written Informed Consent Forms.

## AUTHOR CONTRIBUTIONS

Dongkui Gong and Yin Sun contributed to experimental design, experiment conduction, data analysis, and manuscript editing. Junhua Zheng and Chawnshang Chang contributed to experimental design and manuscript editing. Tzong‐jen Sheu and Wei Zhai contributed to in vivo experiments conduction. Wei Zhai and Changcheng Guo contributed to conducting clinical samples experiments and database analysis.

## AVAILABILITY OF DATA AND MATERIALS

The datasets used during the study are available from the corresponding author on a reasonable request.

## Supporting information

Supporting Information

Supporting Information

Supporting Information

Supporting Information
