# Supplementary material for: Androgen receptor decreases the renal cell carcinoma bone metastases via suppressing the osteolytic formation through altering a novel circEXOC7 regulatory axis
Source: Clin Transl Med. 2021 Mar 24;11(3):e353. doi: 10.1002/ctm2.353 (PMC7989709; doi:10.1002/ctm2.353)
Supplement: Supplementary file 1 — Supporting Information [file CTM2-11-e353-s003.docx]

**Funding information**

National Institutes of Health (NIH) of United States, Grant/Award Number: CA156700; National Natural Science Foundation of China, Grant/Award Number: 81972369.

**Author**

Dongkui Gong^1,2^* ｜Yin Sun^2^* ｜ Changcheng Guo^2,3^ ｜ Tzong-jen Sheu^4^ ｜Wei Zhai^2,5†^ ｜ Junhua Zheng^6†^ ｜ Chawnshang Chang^2,7†^

^1^Department of Urology, The First Affiliated Hospital of Soochow University, Suzhou, Jiangsu, China

^2^George Whipple Lab for Cancer Research, Departments of Pathology, Urology, Radiation Oncology and The Wilmot Cancer Center. University of Rochester Medical Center, Rochester, NY, USA

^3^Department of Urology, Shanghai Tenth People’s Hospital, Tongji University School of Medicine, Shanghai, China

^4^Department of Orthopedics and Center for Musculoskeletal Research, University of Rochester Medical Center, Rochester, NY, USA

^5^Department of Urology, Renji Hospital, School of Medicine in Shanghai Jiao Tong University, Shanghai, China

^6^Department of Urology, Shanghai General Hospital, Shanghai Jiaotong University School of Medicine, Shanghai, China

^7^Sex Hormone Research Center, China Medical University/Hospital, Taichung, Taiwan

**Correspondence**

Wei Zhai, Department of Urology, Renji Hospital, School of Medicine in Shanghai Jiao Tong University, Shanghai, China. E-mail: jacky_zw2002@hotmail.com

Junhua Zheng, Department of Urology, Shanghai General Hospital, Shanghai Jiaotong University School of Medicine, Shanghai 200080, China. E-mail: zhengjh0471@sina.com

Chawnshang Chang, George Whipple Lab for Cancer Research, Departments of Pathology, Urology, Radiation Oncology and The Wilmot Cancer Center. University of Rochester Medical Center, Rochester 14642, NY, USA. E-mail: [Chang@urmc.rochester.edu](mailto:Chang@urmc.rochester.edu)

* Dongkui Gong and Yin Sun contributed equally to this work.

^†^ Wei Zhai, Junhua Zheng and Chawnshang Chang jointly supervised this work.
