# Supplementary material for: Androgen receptor decreases the renal cell carcinoma bone metastases via suppressing the osteolytic formation through altering a novel circEXOC7 regulatory axis
Source: Clin Transl Med. 2021 Mar 24;11(3):e353. doi: 10.1002/ctm2.353 (PMC7989709; doi:10.1002/ctm2.353)
Supplement: Supplementary file 2 — Supporting Information [file CTM2-11-e353-s002.pdf]

Supplemental Figure 1

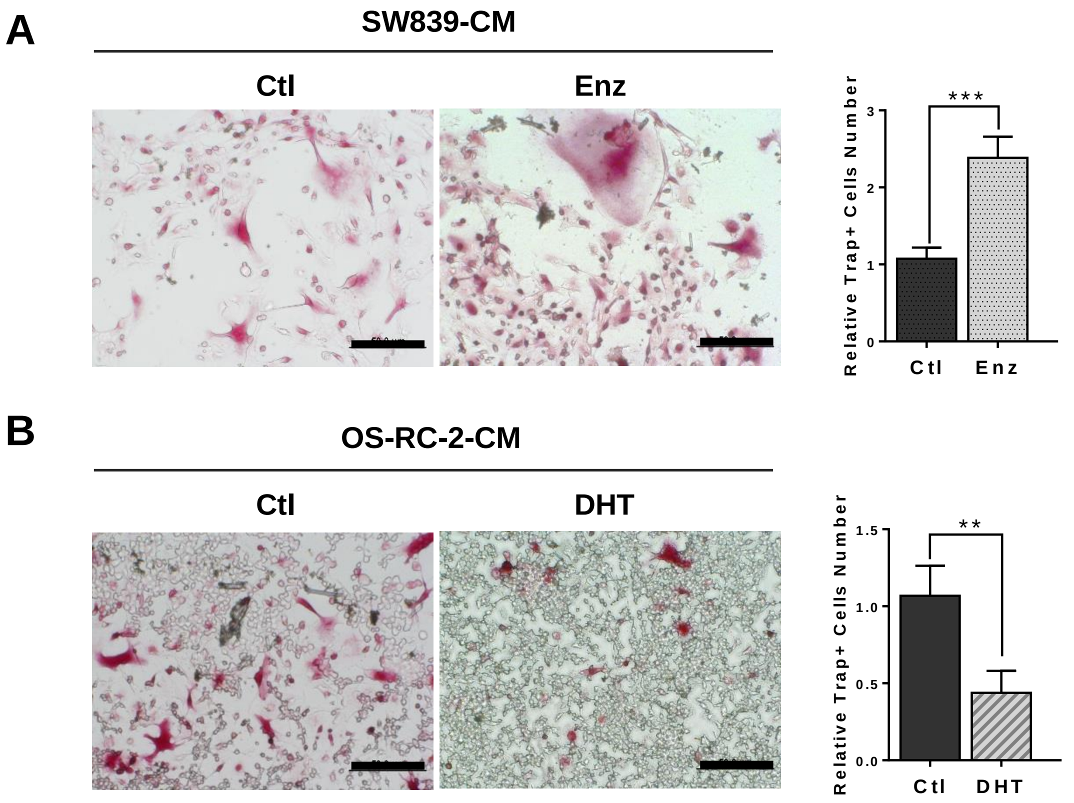

Supplemental Figure 1 (A) BMMs treated with CM from Enz-treated SW839 cells led to more differentiation to TRAP-positive multinucleated osteoclasts. **(B)** BMMs treated with the CM from DHT-treated OS-RC-2 cells displayed less osteolytic formation.

Supplemental Figure 2

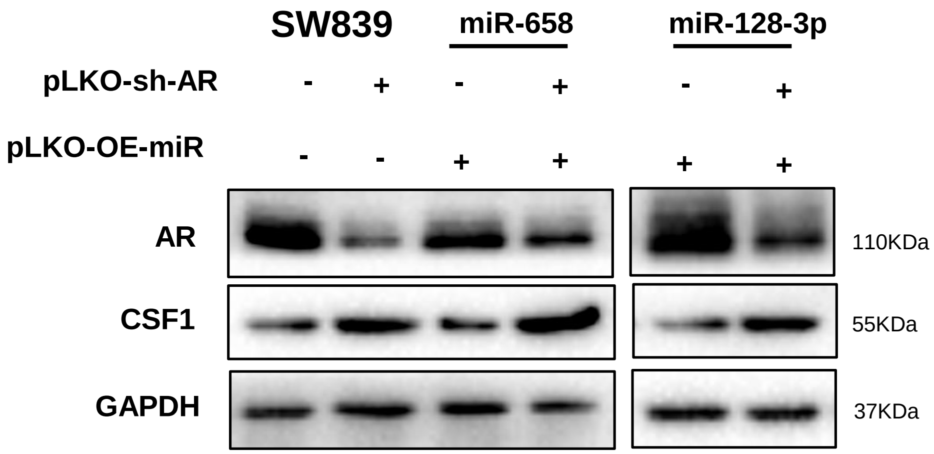

Supplemental Figure 2 WB for miRNA rescue assay revealed that suppressing AR led to increase the CSF1 expression, which could not be reversed *via* adding mimic miRNA **miR-658** or **miR-128-3p** in SW839 cells.

Supplemental Figure 3

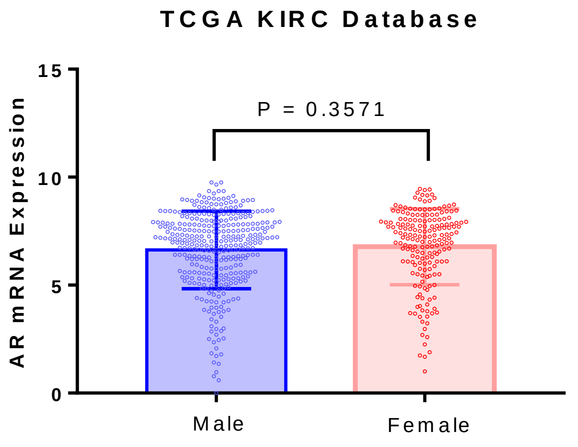

Supplemental Figure 3 AR level has no difference between male (6.628 ± 0.09701, n=345) and female (6.777 ± 0.1286, n=188) (P = 0.3571) tumors in KIRC dataset (n=533).
