# Supplementary material for: Androgen receptor decreases the renal cell carcinoma bone metastases via suppressing the osteolytic formation through altering a novel circEXOC7 regulatory axis
Source: Clin Transl Med. 2021 Mar 24;11(3):e353. doi: 10.1002/ctm2.353 (PMC7989709; doi:10.1002/ctm2.353)
Supplement: Supplementary file 3 — Supporting Information [file CTM2-11-e353-s004.pdf]

Table S1 Primers, Probe and RNA sequences

qRT-PCR primers	
Human MCSF forward	GAAGGAGGACCAGCAAGTG
Human MCSF reverse	GTTCCACCTGTCTGTCATCC
Human IL-8 forward	CGGCGACGACCCATTCGA AC
Human IL-8 reverse	GAATCGAACCCTGATTCCCCGTC
Human RANKL forward	GTCGCCCTGTTCTTCTATTTC
Human RANKL reverse	TCTGCTCTGATGTGCTGTG
Human IL-6 forward	GACAGCCAACTACGATGATG
Human IL-6 reverse	GCAAGTCTCCTCATTGAATCC
Human RUNX2 forward	ACCACTCACTACCACACCTAC
Human RUNX2 reverse	CTTCCATCAGCGTCAACACC
Human MMP13 forward	CCTTGATGCCATTACCAGTC
Human MMP13 reverse	AACCTTCCAGAATGTCATAACC
Human PTHrP forward	TTACGGCGACGATTCTTCC
Human PTHrP reverse	CAGTCACTCCAGAGTCTAACC
Human β-actin forward	CATCCTGCGTCTGGACCT
Human β-actin reverse	GTACTTGCGCTCAGGAGGAG
Human GAPDH forward	GGA GCG AGA TCC CTC CAA AAT
Human GAPDH reverse	GGC TGT TGT CAT ACT TCT CAT GG
Human ADAR F	CTG AGA CCA AAA GAA ACG CAG A
Human ADAR R	GCC ATT GTA ATG AAC AGG TGG TT
QIK F	AAG CCC ACC CCA GAT TAC CT
QIK R	ACT CTG CTA ATT TCT TCG TCC AG
ADAR2 F	GTG AAG GAA AAC CGC AAT CTG G
ADAR2 R	CAG GAG TGT GTA CTG CAA ACC
DHX9 F	ACA CAG GTT CCC CAG TTC AT
DHX9 R	ATA CTG GCA TGA GGA CGA GG

Probes	
circEXOC7	5'-TTATCTGCGTAACTCTCCAC-3'

ChIP primers	
Primer1 forward	CCAGGTTCAAGCAATTCTCC

Primer1 reverse	AAGACCAGGTGTGGTGGTTC
Primer2 forward	ATGTCTTCTAGGCCCCTGCT
Primer2 reverse	TTGTTCCAGGGAATCCAGAC
Primer3 forward	TTACCTTCATGGGACGCACT
Primer3 reverse	CTAACGCAGGTCGCTCCTT

**Luciferase assay primers**	
CSF1 3’UTR psicheck	
CSF1 F:	CAGTAATTCTAGGCGATCGCagggaattctaagctggacg
CSF1 F-del	CAGTAATTCTAGGCGATCGCccatcctcctggaatgtggt
CSF1 R:	AGATATTTTATTGCGGCCAGCggcagcttgtgcacttcttt
pgl3 DHX9 gibson F	CTA TCG ATA GGT ACC GAG CTG TAC AGA CAG GGT TTT GCC ATG TTG GCC AGG
pgl3 DHX9 gibson R	CCA AGC TTA CTT AGA TCG CAT GCG CAG AGC GCG TGC CAG G
pgl3 DHX9 del ARE internal F	AAG AAA ATA ATA AGT AAA TGA TAA AC
pgl3 DHX9 del ARE internal R	GTT TAT CAT TTA CTT ATT ATT TTC TTC TTA ATT AAG AAT AAA ATC ATT G

KEY RESOURCES TABLE

REAGENT or RESOURCE	SOURCE	IDENTIFIER
**Antibodies**		
**Normal rabbit IgG**	Santa Cruz Biotechnology	sc-2027
**AR Antibody (N-20)**	Santa Cruz Biotechnology	sc-816
**DHX9-DExH-Box Helicase 9**	Novus Biologicals	NB110-40579
**M-CSF antibody**	GeneTex	GTX18645
**EXOC7-Exo70 Antibody (ZZ-7)**	Santa Cruz Biotechnology	sc-100733
**IL-6 Antibody (E-4)**	Santa Cruz Biotechnology	sc-28343
**AGO2-eIF2C2 Antibody (4F9)**	Santa Cruz Biotechnology	sc-53521
**GAPDH (6C5)**	Santa Cruz Biotechnology	sc-32233
**Experimental Models: Cell Lines**		
**OS-RC-2**	NICLR http://www.cellresource.cn/fdetail.aspx?id=292	3111C0001CCC000292
**SW839**	ATCC	ATCC® HTB-49™
**786-O**	ATCC	ATCC® CRL-1932™
**Caki-1**	ATCC	ATCC® HTB-46™
**ACHN**	ATCC	ATCC® CRL-1611™
**HEK293T**	ATCC	ATCC® CRL-1573™
**Oligonucleotides**		
**CSF1 3’UTR psicheck**	This paper.	See Table S1
**pgl3 DHX9 gibson**	This paper.	See Table S1
**pgl3 DHX9 delete ARE**	This paper.	See Table S1
**Probes**		
**circEXOC7**	This paper.	See Table S1
**Plasmid**		
**pMD2.G**	addgene	12259
**psPAX2**	addgene	12260
**pBSK**	addgene	67504
**pLKO.1 puro**	addgene	8453
**pWPI**	addgene	12254
**pGL3-Basic**	Promega	E1751
**psiCHECK2**	Promega	C8021
**Software and Algorithms**		
**GraphPad Prism 7**	GraphPad Software	https://www.graphpad.com
**Microsoft Office 2013**	Microsoft	https://www.microsoft.com/fr-fr/
**Image-pro Plus**	Media Cybernetics	https://www.mediacy.com/imageproplus
**SPSS 23.0**	IBM Inc	https://www.ibm.com/support/pages/downloading-ibm-spss-statistics-23

Table S2 Epidemic Characteristics

Gender	Number	Proportion
**male**	825	76.7%
**female**	251	23.3%
total	1076	100.0%

Subtypes	Number	Proportion
**ccRCC**	988	91.8%
**pRCC**	29	2.7%
**chRCC**	45	4.2%
**Others**	14	1.3%

	Total	Male	Female
**ccRCC**	988	763	225
**Bone Meta**	22	14	8

ccRCC:clear cell renal cell carcinoma

pRCC:papillary renal cell carcinoma

chRCC:chromophobe renal cell carcinoma

Table S3 IHC quantification analysis

	n	IOD		*P* value
		Mean	SD	
**Adjacent tissues**	26	56483	19639	
**Primary tumor**	39	102198	31678	<0.001*
**Bone metastasis**	20	24630	11023	<0.001*

IOD:Integral Optical Density. 400X microscrope view

*Statistically significant.

P value from one-way ANOVA analysis.

Table S4 Top 32 circRNAs information

		circRNA	Alias	GeneSymbol	Length	F	R	criterion
	1	hsa_circRNA_051641	hsa_circ_0051641	AP2S1	1413	GAGAGACCAGCCAGACGAAG	CAGCATAGCGGCGGTAAATG	
	2	hsa_circRNA_400037	hsa_circ_0092355	EXOC7	200	TGCTACTTGAAAACTACCAGCC	AACAGCACAGCTATGACCTTGA	
	3	hsa_circRNA_103551	hsa_circ_0068563	XXYLT1	148	CCATCTTCTTCCTCTCGGTCG	CTGAAGTGCTTCTGCATGGC	
	4	hsa_circRNA_000361	hsa_circ_0001275	PLCL2	249	GCCTAGTCTAAGGAGGTGTGTG	TCCTCCCAATGGTGAGCAAG	
	5	hsa_circRNA_003494	hsa_circ_0003494	ORC1	1819	GAAACCCGTGAGAGCCAAGA	ACCCGAACAAGGCCAATGAT	
	6	hsa_circRNA_405974		ACVR1	2716	ATGGGCTCAGACTCCCAAAC	CAGGGTGCAATTCTGGTCTCT	
	7	hsa_circRNA_406587		TRIO	444	GGTCTTGATCCTCCTGACAGC	AATGCCTTTTCCCTGCTTGAC	
UP	8	hsa_circRNA_406430		RP11-440L14	642	TCAGGGTTCCTCAGGGGATT	AAGTGTGTAAGGACCGCCTG	Control group expression>190, FC>3.88, Length<4000
	9	hsa_circRNA_102207	hsa_circ_0045881	AFMID	254	AAGCCTTGCCTTTCTTCCTGT	CGTCGGGGAAGTAAATGTCCA	
	10	hsa_circRNA_406326		QTRTD1	2936	CCTGACAGCGAGAGTTGCTT	CCTTCCCTGCATCCCTTGAG	
	11	hsa_circRNA_020845	hsa_circ_0020845	TSSC2	1372	GGGAGTATTACAGCCGCCTC	CACAGACGAGAGAAGGAAGGT	
	12	hsa_circRNA_001876	hsa_circ_0001876	STX17	239	ATGCTGCAGAATCGTGGGAA	TCTGAGAACTAGCTTCAGCTTCA	
	13	hsa_circRNA_005108	hsa_circ_0005108	FBXO33	1337	TGAGCATTGGAAAGCCCTGT	GGTCCACCTTGCTGAGGTATG	
	14	hsa_circRNA_104600	hsa_circ_0005927	VDAC3	1675	TGCTTGGACAGCTGGGAGTA	GGAAGTCCGCAGCCTTGTAA	
	15	hsa_circRNA_069714	hsa_circ_0069714	DCUN1D4	72	TATTCATAAGATCTACCACACCC	TTGCCAGTGTTGAGAGATGAGA	
	16	hsa_circRNA_104152	hsa_circ_0077248	SNX14	238	TGCTACTTGAAAACTACCAGCC	AACAGCACAGCTATGACCTTGA	
	17	hsa_circRNA_001831	hsa_circ_0001831	SCRIB	571	TCAGGATCCTCCCCTGTGTT	TAGGGTCCTCCCGAAGTACG	
	18	hsa_circRNA_086474	hsa_circ_0086474	RPS6	2664	CGCCAGGAACAAATTGCGAA	CCTGCTTCATGGGGAAACCT	
	19	hsa_circRNA_091743	hsa_circ_0091743	BGN	3685	GCGCAGATGACACCATCAAC	CAGTTCATGTTCCGGAGCCC	
	20	hsa_circRNA_101835	hsa_circ_0005615	NFATC3	1136	CCCTTTACCTGGAGCAAACCA	TGGATGCACAATCATCTGGCT	
	21	hsa_circRNA_030162	hsa_circ_0030162	TPT1	3017	ATCCAGATGGCATGGTTGCT	CGGAGGCATTTCCACCAATG	
	22	hsa_circRNA_105039	hsa_circ_0091934	FLNA	2800	CAGGCAAAGGCAAAGTGACG	CTCGTCACCACCGTACTTGA	
	23	hsa_circRNA_074530	hsa_circ_0074530	CD74	502	GCACCATTGGCTCCTGTTTG	GTACACCTTCAGGGGGTCAG	
	24	hsa_circRNA_101460	hsa_circ_0034067	CYFIP1	91	ATACATTGAACAAGCCACCGTC	TGCATTTCTGTCTTCAAAGTTAGT	Treatment group expression>100, ratio>3.8, FC>5 Length<4000
DOWN	25	hsa_circRNA_057748	hsa_circ_0057748	CFLAR	3676	ACCAGTGAAGAAATCCATTCAG	GAGCGCCAAGCTGTTCCTTA	
	26	hsa_circRNA_104032	hsa_circ_0075303	CANX	115	CGTACCTGATCCAGACGCAG	TTCGGGTTTTGTGGCCTCTT	
	27	hsa_circRNA_101050	hsa_circ_0003770	ARF3	1535	GCAATGATCGGGAGCGAGTA	GCAGTGGCAGTCTGGTTTTG	
	28	hsa_circRNA_007250	hsa_circ_0007250	PDIA6	1409	TGTGATTGAGCTGACAGACGA	CACTCAGCGCAGCATCTACA	
	29	hsa_circRNA_104597	hsa_circ_0084021	PLEKHA2	2434	GACTGGGTTGAAGCCCTGAA	TTGGTTTCTGTTTTGGGGTAGC	
	30	hsa_circRNA_103670	hsa_circ_0006168	CNOT6L	3313	TTGGTCGGCTCTTCCAGCTA	CCCATTGGCTACCTCCTCTG	
	31	hsa_circRNA_102231	hsa_circ_0046263	P4HB	446	CCAACAGTGACGTGTTCTCCA	TCACGATGTCATCAGCCTCTC	
	32	hsa_circRNA_002082	hsa_circ_0002082	MALAT1	868	TACTGTTCTGATCCCGCTGC	AGGGCCTCTATTGCCATGTG	
