# Supplementary material for: Androgen receptor decreases the renal cell carcinoma bone metastases via suppressing the osteolytic formation through altering a novel circEXOC7 regulatory axis
Source: Clin Transl Med. 2021 Mar 24;11(3):e353. doi: 10.1002/ctm2.353 (PMC7989709; doi:10.1002/ctm2.353)
Supplement: Supplementary file 4 — Supporting Information [file CTM2-11-e353-s001.docx]

Supplementary Materials

Trap+ Staining Protocol

1. Discard culture media by pipette, wash with PBS X 1 time  (not medium)

2. Fix with 10% Formalin 100µl at RT for 10-30 mins

3. Wash with PBS X 3 times

4. Refix with 95% Ethanol: Acetone (1:1) at RT for 1 min

5. Air dry for 10 mins

6. Stain cells with Trap staining solution for 30 mins at 37℃

7. Wash with H_2_O

Alcian Blue/Orange G & TRAP+ staining

1. Deparaffinize slides and hydrate to 70% and AIR DRY.

2. Place in Acid-alcohol for 30 seconds and drain briefly on paper towel (DO NOT

RINSE).

3. Place in Alcian Blue Hematoxylin for 25 minutes

4. Wash gently in distilled water for 4 changes

5. Differentiate in Acid-alcohol for 3 seconds

6. Rinse gently in distilled water with 3 changes

7. Place in 0.5% Ammonium water for 15 seconds

8. Rinse in distilled water with 2 changes

9. Place in 95% EtOH for 1 minute (DO NOT RINSE)

10. Place in Eosin/Orange G for 1 min 30 sec

11. Stain with Trap staining solution for 30 mins at 37℃

12. Dehydrate with 3 changes of 95% EtOH and 2 changes of 100% EtOH (1 minute

per change)

13. Clear with 3 changes of xylene and place on coverslip

RESULTS:

Osteoclasts ------------------------------------------------- pink to red

Bone/Cartilage ------------------------------------------- pale blue to blue

Trap staining buffer (500 ml) :

100mM NaOAc.3H2O 6.8g

50mM NaTartrate.2H_2_O 5.8g

Acetic acid 1~1.1 ml

Adjust pH at 5.0

Trap staining solution

1. 5mg AS-MX (sigma, N4875) in 250 µl EGME (Ethylene Glycol Monoethyl Ether)

2. 30mg Fast Red Violet LB salt (Sigma, F3381) in 50 ml Trap staining buffer

3. Put 1 and 2 together and mix well
